# Thread fragrant moxibustion for a young women with atypical hand herpes zoster: A case report

**DOI:** 10.1097/MD.0000000000041169

**Published:** 2025-01-03

**Authors:** Yi-Mei Zhang, Yong-Cheng Wang, Xing-Jun Han, Zhi-Wei Xu, Xing-Lin Zhao, Xiao-Fen Yuan, Wen-Xiu Wang, Hong-Ling Jia

**Affiliations:** a Second School of Clinical Medicine, Shandong University of Traditional Chinese Medicine, Jinan, China; b Department of Acupuncture and Moxibustion, Second Affiliated Hospital of Shandong University of Traditional Chinese Medicine, Jinan, China; c Department of Cardiovascular, Affiliated Hospital of Shandong University of Traditional Chinese Medicine, Jinan, China.

**Keywords:** atypical, hand, herpes zoster, thread fragrant moxibustion

## Abstract

**Rationale:**

Hand herpes zoster (HHZ) is a very rare skin disease in clinical practice. Due to the lack of specificity in its early clinical manifestations, HHZ is easily misdiagnosed as hand eczema or other lesions. Herein, we present a case with atypical HHZ.

**Patient concerns:**

A 25-year-old woman experienced left palm pain accompanied by upper limb stabbing pain, general weakness, and low-grade fever after staying up late. At first, she was diagnosed with peripheral nerve compression and hand eczema, and received relevant symptomatic treatment, but her condition did not improve. Diagnosed with herpes zoster on the hand after skin biopsy, treated with thread fragrant moxibustion (TFM).

**Diagnoses:**

The HHZ diagnosis was established based on clinical appearance and dermatological findings eventually.

**Interventions:**

The intervention project was TFM treatment once a day in the acupuncture and moxibustion department, and the whole treatment period lasted for 10 days.

**Outcomes:**

After 3 more TFM treatments were given, the burning sensation and itching in her palms as well as the pulsatility of the finger blood vessels had significantly reduction, the visual analog scale scores significantly decreased. After 10 treatments, the patient’s herpes gradually falls off, revealing pink new skin without any discomfort.

**Lessons:**

Herpes zoster can appear in any part of the human body with different clinical manifestations. Herpes zoster in the hands is very rare in clinical practice, and it is often misdiagnosed as hand eczema or other lesions in the early stages of the disease. The case is not only highlights the challenging diagnosis in the absence of a characteristic rash at the initial stage but also confirmed the definite therapeutic effect of TFM on herpes zoster.

## 1. Introduction

Herpes zoster (HZ) is a disease in which latent varicella zoster virus is reactivated in the dorsal root ganglion, resulting in the distribution of herpes on the skin innervated by the relevant neurons.^[1]^ The lesions consist of a group of vesicles or bullae, typically located on the trunk or face, and are associated with pain in the affected area.^[2]^ Herpesvirus related infections affect 60% to 95% of adults.^[3]^ In the acute eruptive phase, multiple umbilicated and painful vesicles develop with the characteristics often manifest as often burst, ulcerate, and eventually dry out and the strongest infectivity because they contain higher virus titers. Pain is often accompanied with pruritus, decreased sensation, and allodynia within the affected dermatome(s). In more than 90% of cases, pain precedes the skin eruption by days to a week.^[4]^ The efficacy of antiviral therapy is maximized when initiated within the first 72 hours of disease onset.^[3]^ While unresponsive to nonsteroidal pain medications. The phase may last 2 to 4 weeks while pain can continue longer.^[4]^ HZ can appear in any part of the human body with different clinical manifestations, while head, neck, and trunk are the most affected sites.^[5,6]^ It is often misdiagnosed as hand eczema or other lesions in the early stages of the disease. Additionally, herpes zoster on the hands is very rare. The case is not only highlights the challenging diagnosis in the absence of a characteristic rash at the initial stage. Moreover, improper or untimely treatment can easily lead to postherpetic neuralgia, etc.

In many previous clinical studies and trials, thread fragrant moxibustion (TFM) has effectively treated HZ.^[7–9]^ We present a case of a young female nurse with no previous underlying disease as well as no medical history of skin illness and family genetic history who developed hand herpes zoster after working several consecutive night shifts. TFM was chosen and its therapeutic effect on herpes zoster was observed. After full communication with the patient, the patient signed an informed consent form for the publication of the case.

## 2. Clinical report

A 25-year-old female presented to the emergency department with thermalgia in the left palm accompanied by upper limb tingling, and generalized asthenia accompany low-grade fever that started 3 days prior to presentation. Originally, orthopedic physician who diagnosed her as peripheral nerve compression syndrome and treated her with supportive measures(ibuprofen and aescuven forte). On the following day, her left palm developed extremely itchy and burning pain in her left palm accompanied by significant scattered pulsation, including palm, interphalangeal, and fingertip areas, especially where there is a noticeable sense of vascular pulsation at the fingertip which seriously affects normal life and work. Therefore, she immediately presented to the emergency department to exclude drug allergies or poisoning reactions due to her allergic constitution.

**Figure 1.**
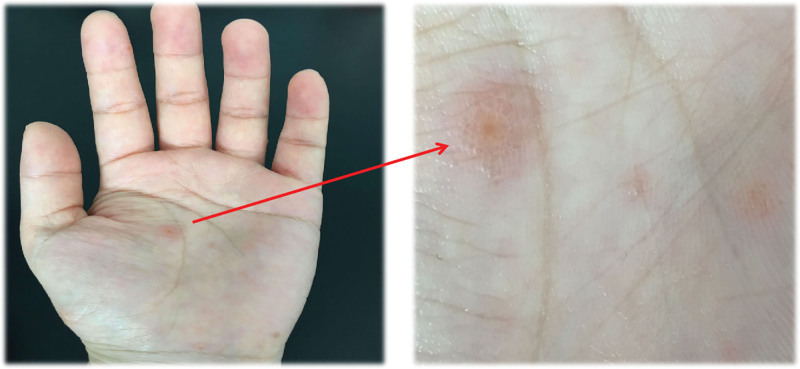
Initial palm herpes.

Upon examination, there were no obvious abnormalities in vital signs. Her left upper limb skin showed no abnormalities such as erythema or rash, and the stinging sensation disappeared. However, integumentary examination revealed visible scattered erythema and clustered blisters on the palmar surface, finger surface, and distal of the index finger in the left hand, with thick blister walls, clear blister fluid, and basal erythema. Those with large blisters are like soybean, while those with small blisters are like millet grain meanwhile itching and burning pain gradually worsen (Fig. 1).

Inspection results showed that there are no significant abnormalities in the complete blood count test are normal as well as routine urinalysis (‐), liver and kidney function tests (‐), blood glucose (‐), human immunodeficiency virus (‐). Her symptoms were ruled out as drug-related issues in the emergency department, and a professional dermatologist was invited to provide consultation support. The dermatologist gave a preliminary diagnosis was hand eczema thus recommended applying triamcinolone acetonide econazole cream to the affected area, once in the morning and once in the evening. And take loratadine orally once a day, 1 tablet at a time (10 mg).

After 2 days, there was no improvement in symptoms in the left hand, and clustered blisters became more prominent and gradually fused. Dermatologists found that the clinical characteristics of herpes zoster were more pronounced and verified by local biopsy on the lesion. At the same time, the doctor suggested that she has to take medication such as acyclovir tablets for treatment, while the patient refused and sought for the Traditional Chinese Medicine treatment to relieve her pain and anxiety as soon as possible. Therefore, she sought further treatment in the acupuncture and moxibustion department.

The patient was first assessed by the acupuncture and moxibustion doctor before treatment, including subjective symptoms evaluated by visual analog scale such as itching, pain and vascular pulsation, as well as objective symptoms by Eczema Area and Severity Index scores such as the size of erythema and the number of papules.

**Figure 2.**
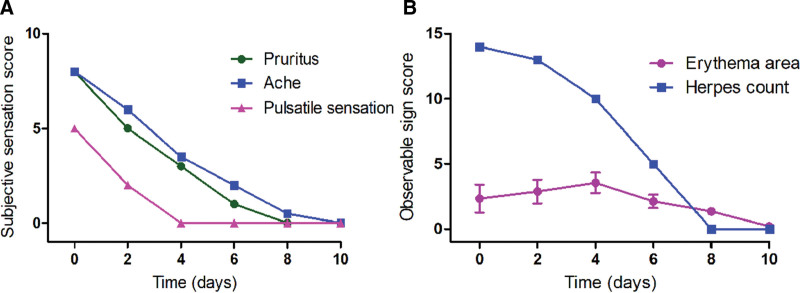
The subjective and objective symptom scores of patient of patient before and after treatment. (A) Image showing that the subjective symptoms of the patient include pruritus, ache, and pulsatile sensation scores were lower than before treatment. (B) Image showing the scores of objective symptoms of the patient include the area of erythema and the number of herpes were decrease.

**Figure 3.**
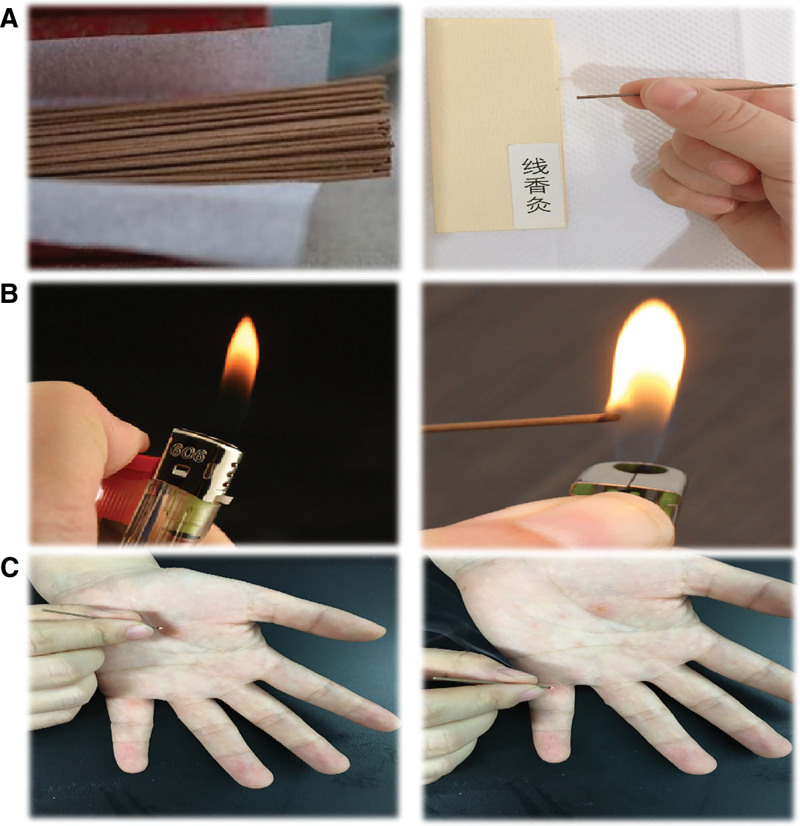
TFM operation steps. (A) Image showing holding posture. (B) Image showing ignite thread fragrant. (C) Image showing thread fragrant moxibustion therapy. TFM = thread fragrant moxibustion.

According to the patient’s description, the patient’s left hand had the highest pain score of 7.5 points, itching score of 8.5 points, and blood vessel pulsation score of 5 points (Fig. 2). After understanding the specific situation of this patient, we gave her treatment with TFM. The specific operation process is as follows. First, prepare a traditional Chinese medicine incense stick (Fig. 3A). Then, the doctor pinches one end of the incense stick with his right thumb and index finger, exposing one end about 1 cm for ignition (Fig. 3B). Finally, shake off the burning flame, and when it forms a bead shaped spark, quickly press the bead shaped charcoal spark head directly onto the blister (Fig. 3C). Once pressed, immediately leave which is called one zhuang. Generally, large blisters should be treated 5 to 7 zhuang, and small rash spots should be treated 2 to 3 zhuang. Treatment should be given once a day, with 10 times serving as a course of treatment.

**Figure 4.**
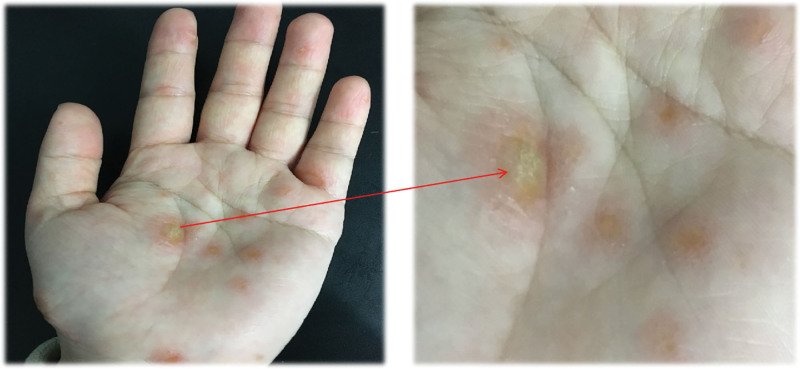
Changes in erythema and herpes on the 6th day of treatment.

**Figure 5.**
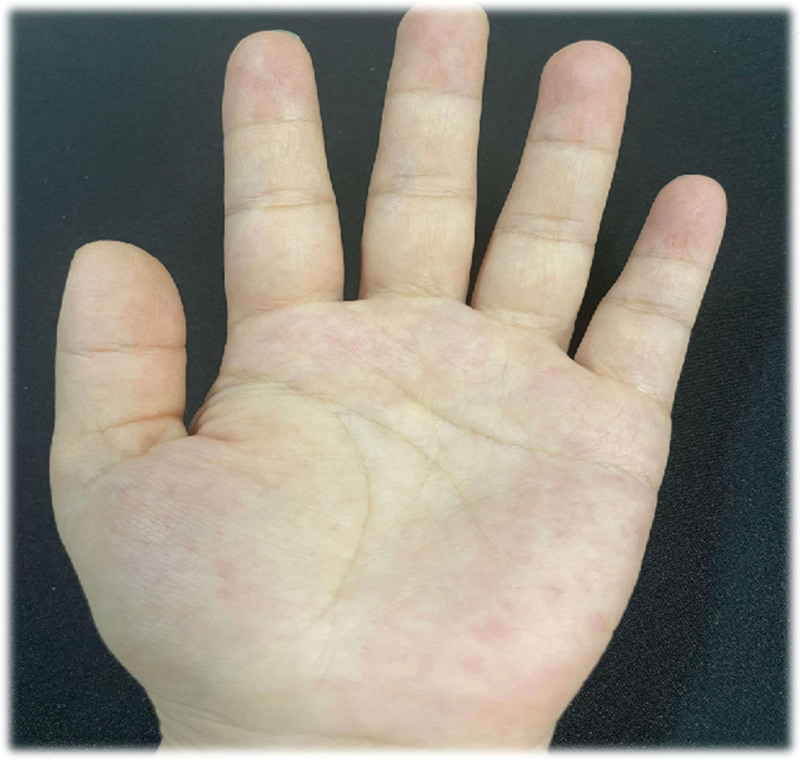
Left hand performance during follow-up after 1 month of treatment.

Therefore, she received TFM treatment once a day in the acupuncture and moxibustion department, and assessed the changes of symptoms before and after treatment every 2 days, and made records. On the 4th day of TFM treatment, she told that the burning sensation and itching in her palms had significantly decreased, and the pulsatility of her finger blood vessels had basically disappeared, with the visual analog scale scores significantly decreased (Fig. 2A). After 6th treatments, the tissue fluid inside the blister is gradually absorbed, and the skin covering the blister has separated from the bottom of the palm skin. After sixth treatments, the skin covering the blisters has peeled off from the bottom of the palm skin. Meanwhile, the top of the large blisters gradually turns yellow, scabs and hardens, and the peripheral redness and halo of all blisters increase (Figs. 2B and 4). At this point, all subjective discomfort has basically disappeared. Four more TFM treatments were given, as a result, the patient’s herpes gradually falls off, and pink new skin appears at the base of the herpes, slightly shallower than the surrounding normal skin without any discomfort. We shown off the symptom changes during her entire TMF treatment period based on her symptom rating (Fig. 2). No adverse events occurred throughout the entire treatment period. After a course of treatment, the patient received a follow-up from the doctor who treated her with TFM. Currently, there are no discomfort symptoms and returned to normal in her left hand, and took photos to gave feedback (Fig. 5). Written informed consent was obtained from the patient for publication of this case report and accompanying images, and all procedures involving the patient were conducted in accordance with the ethical standards of the Ethics Committee of the Second Affiliated Hospital of Shandong University of Chinese Medicine.

## 3. Discussion

HZ is caused by reactivation of latent varicella zoster virus infection within the sensory nerve ganglion of the spinal or cranial nerves.^[10]^ Subsequently, spreads along the peripheral nerves to the skin, leading to obvious complications such as burning pain, severe itching, and papules in the affected skin area, especially improper treatment or misdiagnosis can easily cause postherpetic neuralgia, which brings a very painful experience to patients. It is on account of the nerve tissue damage caused by the outbreak of herpes zoster, inflammatory substances such as substance P, histamine, and bradykinin are released, which promote ectopic discharge of C fibers and lead to peripheral sensitization.^[10]^

HZ can affect people of all ages and cause a health burden,^[11]^ the diagnosis is usually made on the history and examination findings. The most common affected areas are the chest and waist skin areas. The virus invasion of the median, radial, and ulnar nerves is relatively rare, particularly in cases where skin lesions are limited to the hands. In the early stages of the disease, there are often only blisters, making it easy to misdiagnose as hand eczema. Study had shown that the initial stage of skin injury is the erythematous macula phase, followed by a vesiculopapular phase that appears within 1 to 2 days and continues to occur for the next 3 to 4 days.^[5]^ At this point, the typical clinical manifestation of HZ is evident. The cornerstone treatment incorporate the application antiviral therapy and analgesics including acyclovir and famciclovir effectively decreases acute pain, promotes lesion healing and prevents PHN. The effects of antiviral therapy are most favorable if given during the viral replication time which is ≤72 hours after onset of the skin rash. Nevertheless, it remains a controversy whether antiviral treatment beyond the 72 hours of the onset of rash is beneficial.^[12]^ Obviously, the patient in our case study presented beyond the 72 hours window. In addition, eradication of the virus itself is difficult, for drug-refractory cases, effective treatment is an attractive choice.^[13]^

TFM belongs to a type of moxibustion therapy, which also has the characteristics of comprehensive biological effects such as fire needles and traditional Chinese medicine. It uses the warm stimulation produced by the burning on the body surface of the relevant meridian points, so as to achieve the therapeutic effect of clinical diseases.^[14,15]^ While the precise mechanism underlying the effect of TFM therapy on HZ on the hands has yet to be fully validated, although several possible mechanisms have been considered. Firstly, moxibustion can inhibit TRPV1 expression and release of histamine receptors, thereby improving itching symptoms.^[15]^ Secondly, TFM can significantly reduce the content of substance P in peripheral serum, decrease the transmission of stimulating pain sensation, inhibit the release of inflammatory mediators, and alleviate persistent nerve damage.^[9]^ Thirdly, TFM alleviates pain by stimulating secretion of anti-inflammatory factor IL-10 and reducing hyperalgesia at nerve injury part.^[16]^ Finally, TFM improves pain may related to change the protomics indexes like MFG-ES, lymphotoxin beta/TNFSF3, IL-19, neuritin, NCAM-1/CD56, and PECAM-1/CD31.^[17]^ The warm effect of gathering can promote microcirculation in the lesion area by regulating cutaneous nerves, which potentially absorb inflammatory mediators and exert neuroprotective effects.^[18,19]^ Furthermore, the high temperature may generate an effect that directly eliminates the microorganisms and achieves anti-inflammatory effects.^[20]^ Moxibustion has fewer side effects compared to pharmacological interventions, making it an attractive option for patients who are unable to tolerate the side effects of conventional pain medications or who are seeking a non-pharmacological approach to pain management.^[21]^

The present case reports the treatment of HZ on the hand with TFM. This innovative study provides a basis for the effectiveness of TFM in the treatment of HZ on the hands and also provides a complementary therapy for HZ on the hands.

## 4. Limitations

This study presents a descriptive and retrospective character and does not delve into verifying the underlying mechanisms of TFM. Further research is required to interpret the mechanisms better. There is only one case reported, resulting in a relatively small sample size. This limits the universality of research results. We will continue to collect more cases of HZ and further investigate the therapeutic mechanism of TFM, especially hand herpes zoster.

## 5. Conclusion

HZ can appear in any part of the human body with different clinical manifestations. HZ in the hands is very rare in clinical practice, and it is often misdiagnosed as hand eczema or other lesions in the early stages of the disease. The most effective treatment for HZ is antiviral therapy during the viral replication time which is ≤72 hours after onset of the skin rash, but it remains a controversy whether antiviral treatment is antiviral effective if not within the specified effective time. The case is not only highlights the challenging diagnosis in the absence of a characteristic rash at the initial stage but also confirmed the definite therapeutic effect of TFM on herpes zoster.

## Author contributions

**Conceptualization:** Yi-Mei Zhang.

**Data curation:** Yong-Cheng Wang, Xing-Lin Zhao, Wen-Xiu Wang.

**Formal analysis:** Yong-Cheng Wang, Zhi-Wei Xu, Xiao-Fen Yuan.

**Supervision:** Xing-Jun Han, Hong-Ling Jia.

**Writing – original draft:** Yi-Mei Zhang.

**Writing – review & editing:** Yi-Mei Zhang, Hong-Ling Jia.
